# Using communities of practice as a lens for exploring experiential pharmacy learning in general practice: Are communities of practice the way forward in changing the training culture in pharmacy?

**DOI:** 10.1186/s12909-021-03079-8

**Published:** 2022-01-03

**Authors:** Ali M. K. Hindi, Sarah C. Willis, Ellen I. Schafheutle

**Affiliations:** Centre for Pharmacy Workforce Studies, Division of Pharmacy and Optometry; School of Health Sciences; Faculty of Biology, Medicine and Health, The University of Manchester, Oxford Road, Manchester, M13 9PT UK

**Keywords:** Pre-registration pharmacists, Foundation training, General practice, Primary care, Placements, Clinical supervision, Experiential learning

## Abstract

**Background:**

Currently, there is little experiential learning in general practice (GP) during UK undergraduate and postgraduate pharmacy education and training.

**Aim:**

To apply educational theories to explore pharmacy stakeholders’ perceptions of placements in general practice and contribute to the development of a model of experiential learning for pharmacy.

**Methods:**

Qualitative, semi-structured interviews, conducted as part of two studies exploring experiential learning in general practice, with learners and their GP based supervisors. Interviews explored experiences of learning and practice, and what aided or hindered this. An abductive approach to analysis combined inductive coding with deductive, theory-driven interpretation using Lave and Wenger’s concept of “Communities of Practice”.

**Results:**

Forty-four interviews were conducted, with learners and placement supervisors. Participants valued placements for providing authentic patient-facing learning experiences in the workplace, facilitated through legitimate peripheral participation by supervisors and supported by the use of pre- and de-briefing. Learners benefitted from support from their supervisor(s) and other staff during their day-to-day learning (informal learning), whilst also having protected time with their supervisors to discuss learning needs or go through workplace-based assessments (formal learning). Lack of clarity regarding which and how competencies should be assessed / demonstrated in general practice challenged monitoring progress from peripheral to full participation.

Findings suggest that GP placements provide opportunities for learning about the patient journey between care settings; to work effectively with multidisciplinary teams; and consolidation and application of consultation / communication skills learning.

**Conclusions:**

The learning culture of GP supports learners’ development, providing time and opportunities for meaningful and authentic workplace learning, with healthcare professionals acting as supervisors and mentors. These findings can usefully inform implementation of meaningful learning opportunities in primary and secondary care for those involved in pharmacy education and training.

**Supplementary Information:**

The online version contains supplementary material available at 10.1186/s12909-021-03079-8.

## Background

The term "experiential learning” describes work-based learning experiences which bring the learner into contact with others in a particular role and context [1, 2]. Experiential learning is a socio-cultural learning theory where knowledge and meaning are conceptualised as constructed from real-life experience, and as resulting from collaborative engagement within a social environment rather than an exclusively individual process [3].

One of the most common learning theories in medical education is Kolb’s four-stage experiential learning cycle which serves to understand how experience is translated through reflection into concepts, which then guide active experimentation and new experiences [4]. Other experiential learning and socio-cultural theories also highlight the importance of supporting learners to understand and interpret what their experiences mean to them by having: mentorship/guidance from someone more experienced at the workplace, constructive discourse with others, and formal/informal assessments [5].

Medical students experience workplace learning from the start of their undergraduate education [1], and experiential learning theory has informed the design of learning opportunities to meet intended learning outcomes [1]. Research on medical students’ experiential learning highlights the importance of learner workplace integration through active meaningful participation, with staff providing meaningful workplace tasks, and learners supported to reflect on and understand their experiences [6, 7]. In doing so, the workplace context becomes a source of applied knowledge which helps learners understand and carry out their workplace activities effectively rather than superficially to reach their intended learning outcomes [8].

In the U.S. and Canada, experiential learning approaches used in pharmacy colleges commonly involve early exposure of pharmacy students [in the first year or two] to clinical rotations, with a pharmacist preceptor overseeing the process [9, 10]. Reported benefits of such clinical rotations include: direct patient facing experience, increased confidence in communication, and a better understanding of multidisciplinary team working [9, 10]. However, a common criticism of clinical rotations in Canada and US is that they are often relatively short (a few weeks) which often means that students mainly shadow/ observe clinicians with little opportunity for active participation.

Practice-based experiential learning in the 4-year undergraduate Master of pharmacy (MPharm) degree in Great Britain is limited [11, 12], and takes places mainly during 12 months of pre-registration training, traditionally either in community or hospital pharmacy. During the pre-registration training year, trainees have to meet 76 performance standards, set by the pharmacy regulator in Great Britain, against which their tutor signs them off. However, experiences of the pre-registration year is inconsistent and variable across sectors [11, 13]. Whilst all trainees have one pharmacist tutor with overall responsibility, hospital trainees work alongside a range of pharmacists and members of the multi-professional team, whereas in community pharmacy, trainees work mainly with their tutor and a small pharmacy team. Once registered, there is no formal or funded support, and community pharmacists in particular find the transition to independent practice challenging [14].

The National Health Service (NHS) Long Term Plan (2019) [15] envisions a workforce of pharmacists capable of working clinically in general practice (family medicine) and across integrated care pathways [16, 17]. Newly introduced pre-and post-registration placements in general practice offer an opportunity to understand how experiential learning works there, and what these placements contribute to learning and development in pharmacy.

The aim of this paper is to apply educational theories to explore pharmacy stakeholders’ perceptions of placements in general practice and contribute to the development of a model of experiential learning for pharmacy.

### Communities of practice (CoP): the theory

**Fig. 1:**
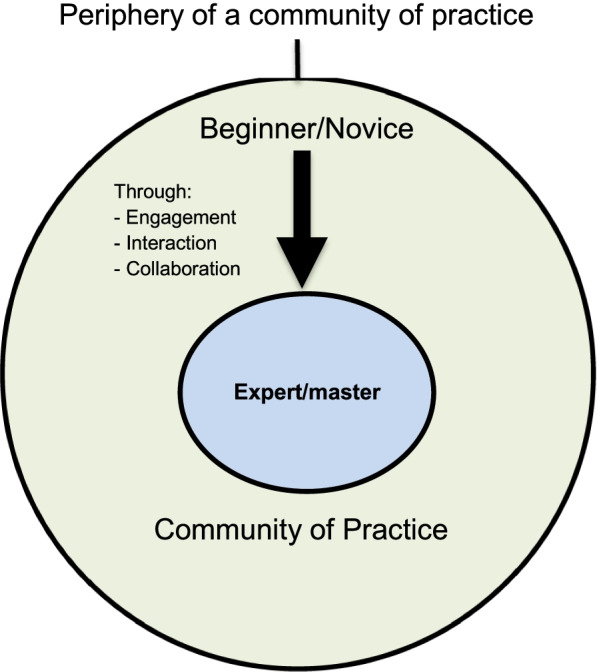
Legitimate Peripheral Participation taken from Lave and Wenger

Lave and Wenger’s ‘communities of practice’ (CoP), which focuses on the relationship between learning and the social situation in which it occurs, provides this paper’s theoretical underpinning [18]. CoP conceptualises that learners participate in ‘communities of practice’ and acquire knowledge and skills through newcomers moving from ‘legitimate peripheral participation’ toward ‘full participation’ in the sociocultural practice of a community (Figure 1). Novices begin by observing and performing basic tasks at the periphery. Through active participation and engagement they become more skilled, assume more responsibility and therefore move into a more central position within the community [18, 19].

Given that learning in CoP is viewed as a series of collective, relational and social processes rather than individual acquisition of knowledge [20, 21] considering pharmacy placements in general practice as CoPs could help to uncover the learning processes that take place within that sector.

### Context

This paper draws on data collected during the evaluation of two publicly funded programmes which involved pharmacy trainees spending time in general practice pre-registration (evaluation 1) and soon after registration (evaluation 2). Both funded programmes sought to investigate possible models of training which provide opportunities for pharmacy learners to develop the knowledge, skills, behaviours and attributes to work across a variety of sectors of care, whilst concurrently gaining a wider understanding NHS system and patient journey. The key elements of each programme are displayed in Table 1. Details on placement structure/organization are provided elsewhere [22, 23]. In this paper, we use the term ‘pre-registration training year’ which was relevant at the time of the study but it is important to highlight that the GPhC recently changed the terminology from ‘pre-registration training year’ to ‘foundation training year’ across Great Britain in the summer of 2021.

**Table 1:** Pre-registration Pharmacists in General Practice Project compared to South East London Foundation Pharmacist Vocational Training Scheme

Evaluation 1. Pre-registration Pharmacists in General Practice Project (2019) [22]	Evaluation 2. South East London Foundation Pharmacist Vocational Training Scheme (SEL FP VTS) [23]
• Around 100 pre-registration trainees were employed in a pharmacy base (community or hospital) and spent between 13 and 26 weeks of their pre-registration training year in general practice.	• Three-year multi-sector programme which involved 8 SEL FP VTS novice pharmacists who spent 6 months in general practice. (Additional file 1)
• Trainees had a GP based pharmacist supervisor as well as their base pharmacist tutor.	• SEL FP VTS pharmacists supported by a general practice based pharmacist supervisor, a general practitioner supervisor and a SEL FP VTS peripatetic education supervisor.
• Pre-registration trainees are not registered healthcare professionals, who even under supervision cannot undertake all pharmacist activities, such as vaccinations	• Novice pharmacists on the other hand are registered healthcare professionals able to practise autonomously, yet still have possible support and learning needs post-registration.
	• Expectations of tasks and scope of practice are higher of novice pharmacists compared to pre-registration trainees.
• Pre-registration trainees and novice pharmacists were funded as supernumerary and enabled to undertake activities that supported them to develop the knowledge, skills and behaviours required of an autonomous general practice based pharmacist.	

Both programmes included completion of a portfolio of evidence to demonstrate competency, and the use of work-based assessments (WBAs) (Table 2).

**Table 2:** Workplace-based assessments (WBAs)

Workplace-based assessment	Definition	Placement
MRCF: Medicines-Related Consultation Framework	The MRCF is a structured validated patient–centred approach to patient consultation. It supports pharmacists in developing consultation skills. This tool gives the opportunity for the supervisor to assess if the pharmacist is an effective communicator and able to shape the patient’s behaviour, through a shared agenda to ensure medicines optimisation.	SEL VTS FP
MRCA: Medication Review and Consultation Assessment	The MRCA has been developed based on the MRCF; it has been adapted to include additional elements relating to medication review and holistic care. The assessment tool is designed to test the range of knowledge, skills and behaviours pre-registration trainee pharmacists are expected to develop in their general practice placement.	Pre-registration placements
Mini-CEX: Mini-Clinical Examination Exercise	A Mini-CEX is used to assess the pharmacy learner’s ability to identify, action and resolve issues effectively when providing pharmaceutical care for a patient. This enables supervisors to review various skills, attitudes, knowledge and behaviours of the pharmacist, and is a useful tool for developing pharmacy staff.	SEL VTS FP & pre-registration placements
CBD: Case Based Discussion	In a CBD the pharmacy learner discusses management and understanding of a case with a supervisor. Within the discussion supervisors are able to probe a learner’s knowledge and approach to dealing with the case.	SEL VTS FP & pre-registration placements
DOPS: Direct Observation of Practice Skills	A DOP assesses the pharmacist’s ability to carry out practical activities. Examples of suitable activities to use a DOPS for are influenza vaccination administration, monitoring of blood pressure or other physical assessment.	SEL VTS FP
Intervention Recording	Intervention recording supports the development of intervention, recommending, justifying and communicating interventions. Examples of when to use: medicines reconciliation intervention, responding to a medication query, medication review, chronic disease review	Pre-registration placements

This paper views work-based learning, support and supervision, through the lens of CoP, to explore what contributes to a ‘successful’ general practice placement – that is, a placement that through practice and participation in a CoP facilitates learner development.

## Methods

### Recruitment

Both funded programmes for novice pharmacists and pre-registration trainees were set up with a request to consent to being contacted regarding an evaluation. The national leads for each of the two programmes provided contact details for learners and their supervisors, following consent to share their details with the research team. The national leads also provided the research team with information relevant to supporting sampling. The research team used a dyad/triad approach which involved the learner and at least one of their tutors being interviewed. Potential participants were emailed invitation letters and participant information sheets, requesting those interested to contact the first author (AH). The first author then arranged an interview. The sample size was determined based on data saturation.

### Data collection

Semi-structured telephone interviews were conducted by the lead author with pre-registration trainees, novice pharmacists, and their general practice based supervisors between January and July 2020. The lead author took field notes during and after the interview. The lead author had considerable experience conducting qualitative research from his PhD work. Interview schedules were informed by existing research [13, 24–27], with questions tailored to understand expectations and experiences of general practice placements, knowledge and skills gained, as well as competence and confidence to apply these. Data collection continued until data saturation was reached.

### Data analysis

All interviews were audio-recorded and transcribed verbatim. Transcription was done via a university approved third party provider. Transcriptions were imported into NVivo 11 to manage the data analysis process. Interview transcripts were analysed by the first author using an abductive approach which integrated inductive data-driven coding with deductive theory-driven interpretation [28] by positioning empirical findings against Lave and Wenger’s concept of Communities of Practice (CoP). Taking a combined iterative and theoretical approach to analysis ensured that CoP was used in an exploratory way to make sense of findings [29]. Analysis and themes were discussed with the co-authors in regular meetings throughout analysis.

## Results

### Participant characteristics

Twenty-two interviews were completed with 14 pre-registration trainees (interview length: 34 - 55 minutes) and their 9 general practice pharmacist supervisors (interview length: 35 - 53 minutes). Twenty interviews were completed with all 8 novice pharmacists (interview length: 25 – 57 minutes) and their 6 general practitioner and 7 pharmacist supervisors (interview length: 15 - 28 minutes).

Using Lave and Wegner’s CoP where each general practice placement is considered a community of practice, findings have been grouped into themes to depict key elements contributing to a ‘successful general practice placement’ (Table 3).

**Table 3:** Summary of themes identified in this study

**Themes**	
**• Welcoming pharmacist learners to the community of practice**: support provided to learners when they first start their GP placement. **• Identity development in the community of practice:** how learners identify their scopes of practice and how their professional identity is influenced by the community of practice. **• The journey from peripheral to full participation:** the process by which learners advance in the workplace by gradually taking on more responsibilities and the role of supervisors in facilitating this progression. **• Formal and informal assessment :** opportunities provided in the workplace for learners to reflect on, and learn from, workplace experiences **• Value of community of practice participation:** main benefits of learners spending time in general practice	

### Welcoming pharmacist learners to the community of practice

According to Lave and Wegner, communities of practice should be inclusive, with new members welcomed and supported. In our studies, most learners felt that their supervisors and other clinical and non-clinical staff were welcoming and accommodating when they arrived. Well prepared sites provided learners with workspace, a structured induction, and (less commonly) staff had a good understanding of the capabilities of a learner. Conversely, the early weeks were challenging for learners where a site was not well prepared and did not offer a formal induction. Learners commonly described the support they received from their supervisors and other clinical/non-clinical staff as making their transition to general practice easier in the early weeks.

### Identity development in the community of practice

The process by which newcomers form a professional identity is directly influenced by the community of practice. In the absence of an established role for pharmacists in general practice, the main challenge encountered by learners was initially identifying their limitations and scope of practice in general practice. Most pre-registration trainees described general practice staff spending considerable time at the start assessing what they could (not) do, because of limited understanding of learners’ capabilities:

*“I don’t know if they knew what they wanted me to do, what they wanted to get out of things. It did kind of feel that I was a missing piece and I didn’t really fit anywhere, but as time went on, obviously they figured out what I can do, what I’m comfortable doing,”. (pre-registration trainee 8)*

Novice pharmacists reported that unlike other placements where the pharmacist role is well-established, they had to be self-directed in seeking learning opportunities.

*“I think it was more just seeing how proactive you need to be in terms of asserting the pharmacist position in the GP practice …if you're not proactive and if you only do the work that just comes to your face, then you don't really make the most out of your role in the practice, patients won't really know you're there. If you're not proactive with your colleagues, then they won't really realise that, you know, they can ask you questions or queries, or anything like that”. (Novice pharmacist 3)*

Understanding learners’ identities and roles posed a challenge for supervisors at the start. Supervisors (mainly GPs) with little experience supervising pharmacy learners had anticipated providing less hands-on supervision and a reduction in their own workload. On the other hand, supervisors with more knowledge/experience of pharmacy recognised the need to focus on getting to know the setting, and the contribution learners could make. Gradually though, supervisors and general practice staff developed a shared understanding of learners’ capabilities.

### The journey from peripheral to full participation

During the first few weeks learners were introduced to policies/procedures, and shadowed clinical and non-clinical staff to understand how general practice worked. Learners then had a flexible monthly plan organised to allow for gradual development of competence and capability:

*“It built up over time. So, initially, I started off with doing reconciling of documentation that came in from hospitals or people requesting prescriptions. And then I also did some work auditing… And then, eventually, I went on to be shadowing pharmacists as they did blood pressure clinics and then I took on my own blood pressure clinic… and then also I’d do some telephone calls, which would be in the morning when patients call up and say, ‘Oh, I need to speak to a doctor’. And then the receptionist would allocate them into pharmacists’ slots to then be called back by a pharmacist. So, I would help with some of the more minor conditions, which would be picked out for me”. (Novice pharmacist 4)*

Supervisors played a key role in engaging learners in their journey from peripheral to full participation. At first, learners needed direct supervision. Supervisors commonly described initially spending considerable time with learners to determine their competence and capability in administrative and clinical tasks.

*“What we [both supervisors] did initially, was that we supervised her [learner] in all the activities before we were confident that she could undertake some of the things on her own. So we didn't let her loose, if you like, at the beginning on any of it”. (SEL FP VTS, supervisor 2, GP)*

As the placement progressed, supervision became more arms-length, with learners working independently when undertaking non-patient-facing activities, querying and feeding back to their supervisors when necessary.

Pre-registration trainees and novice pharmacists took considerable time to build up confidence in patient-facing clinical assessments and medication reviews. Supervisors supported learners to take on a more active role, starting with observing and gradually taking on minor tasks under supervision.

*“We watched the pharmacist do medication reviews and then he’d kind of give us patients that were coming in and research into the problems they might be having; going through their medication list, picking out any kind of health thing we want to do. It was doing what they’re doing but in the prep beforehand, obviously, because we weren’t experienced enough to do it ourselves”. (pre-registration trainee 3)*

Learners observed practitioners using different consultation styles and were able to develop their own approach after spending time with nurses and GPs during clinics.

Over time, learners gradually moved from observation (periphery of the CoP) towards the centre (participation) by providing basic medication reviews for patients with common long-term conditions (e.g. asthma) and basic clinical assessments during clinics (e.g. respiratory rate). Most supervisors described applying an approach similar to that used when supervising undergraduate medical students:

*“We’ve used the same structure as what we would do for the undergraduate medical students…. where he [learner] will see a patient and we’ll protect some time straight after for the supervisor which is myself. Then he’ll see the next patient and then there’ll be some protected time to debrief in front of the patient. So, we’ve used the same for the pre-reg pharmacists and that seems to work really well because then he’s got confidence that if there’s something he’s unsure about, there’s going to be somebody there straightaway for him to handover to”. (Pre-registration placement,: supervisor 7, pharmacist)*

Leadership, mutual trust and respect amongst members are important components of a community of practice. Towards the end of placements, learners felt confident to work independently; supervisors trusted learners’ ability to work without direct supervision.

Novice pharmacists participated in multi-disciplinary team (MDT) meetings, which provided opportunities for shared learning. They perceived discussing cases with other healthcare professionals and learning their approach to a patient case-scenario contributed to their own development.

*“…every day there's a clinical meeting with all the doctors … it's nice to see from a medical point of view how different patients are dealt with and problems are solved. I think that's really good to see for a pharmacist because it gives you a different structure, you can see their thought process and apply it to your own problem-solving methods… it reinforced my consultation skills, it reinforced the way I approached patients”. (Novice pharmacist 3)*

Pre-registration trainees reported very limited opportunities to learn with other healthcare profession trainees, and raised this as an issue for future programme development.

### Formal and informal assessment

Lave and Wegner argue that members of a CoP need both scheduled time and informal opportunities to reflect on, and learn from, workplace experiences. Pre-registration trainees described using self-reflection when writing evidences to demonstrate their competence, and described the value of informal feedback from their pharmacist supervisor. Some novice pharmacists self-assessed their competence based on how much work they completed or the relative ease with which they carried out tasks.

Supervisors supported learners’ development by providing learning opportunities and formative feedback, including regular catch-up sessions. In pre-registration placements, this involved general practice pharmacist supervisors discussing key learning points associated with activities undertaken; asking trainees questions; and getting trainees to read/look up relevant information for self-study (e.g. learning modules, guidelines).

Novice pharmacists had both a GP and pharmacist supervisor. GP supervisors’ offered formal tutorials and shorter, dedicated sessions with protected time for novice pharmacists to discuss queries, specific learning needs or workplace-based assessments. Pharmacist supervisors were more involved in day-to-day training and supervision; they were often the first point of call for ay medication-related queries (e.g. medication management, prescriptions, audits) whilst GP supervisors were reserved for more complex/clinical queries (e.g. physical examination skills, interpreting lab results).

*“When I had my clinics, I would then go to that GP supervisor first, or if she wasn’t available, then I would go to the pharmacist supervisor, or another GP and just discuss the patient with them. And, if it was stuff like medication reconciliation, or something else… then I could also discuss that with the pharmacist, if they were available, if they weren’t busy... So I think personally for my own development, I prefer this style of supervision because it's very individualised, you get protected time with your tutor to go through things, but at the same time, you can always ask anyone for help during the day. It's focused on you and it's focused on your specific development”. (Novice pharmacist 3)*

There were mixed views on the usefulness of workplace-based assessment (WBAs). It was apparent from the interviews that there was a need for guidance on how/when to use WBAs for both pre-registration trainees and novice pharmacists. In pre-registration placements, supervisors either used these tools rarely or not at all**.**

***“****It was a case of just observing other practitioners, seeing what the different styles they used, consultation skills, asking questions when necessary. I thought that was more hands on and […] I wasn’t always looking at the [MRCA] sheet ….” (Pre-registration placement, supervisor 1, pharmacist)*

WBA tools were perceived as beneficial by novice pharmacists when used in conjunction with feedback from supervisors/healthcare professionals.

A common finding from both studies was the need for clarity on competencies that pre-registration trainees and novice pharmacists were expected to demonstrate in general practice.

*“…there’s no competency framework [specific to GPs] for pre-regs, so this is where we struggled a bit. But it’s a case of how many times do you get them to check a temperature or listen to a chest or do a peak flow before you can say that they’re competent to do it on their own” (Site 7, community pharmacy, GP tutor – multiple blocks)*

*“I think the leaders of the programme… need to be quite directive of the standards that are expected and the things that the pharmacist could and should be able to do. …the GPs look for guidance, if you leave it to the GPs, you’ll get huge variation... if the leaders of the programme are clear about what is required and how it should happen, with some flexibility, I think that would improve things moving forward.” (SEL FP VTS, GP supervisor 8)*

### Value of community of practice participation

A successful CoP is determined by the value it brings to its members, and the value the newly formed CoPs brought to members in pre-registration and novice pharmacist placements was apparent. Learners’ consultation and clinical examination skills were viewed as significantly improved, and novice pharmacists reported feeling more able to practise autonomously.

*“with consultation skills, for pharmacists anyway I feel it’s something we don’t do enough of at university. Unlike our medic colleagues who see patients all through their degree, we might do a two week placement here and there but actually when we do consultation skills it’s usually with a member of staff or one of your fellow students as a patient. So you never really develop how to speak to a real person. So I think that’s an important part of what I try and do here is to develop those skills, because I think they’re the ones that we’re missing as pharmacists.”. (pre-registration placements, pharmacist supervisor 3)*

*“That [consultation skills] improved very much… because in a GP practice, patients are coming directly to speak with you, and they’re there only to talk to you and it’s much more personal. And because of the setting, because it’s in a GP’s room, a patient will open up about so many things, and not always stick to one clinical problem…anything can come up basically. So, her [learner] experience developed vastly over the time, over the rotation”. (SEL FP VTS, pharmacist supervisor 4)*

Learners felt they became a useful resource and valued member of the general practice team as their placement progressed. Even pre-registration trainees believed that they contributed to managing some of the general practice workload.

Although novice pharmacists were supernumerary, pharmacist supervisors reported being able to reach clinical targets more quickly. Most GP supervisors valued having an extra member of staff who could help with medicines reviews, audits, meeting performance measures, improving record keeping and liaising with hospital and community pharmacists.

*“Benefits to the practice are huge. I mean it's having an extra member of staff at a difficult time. So having the pharmacist has been hugely beneficial to the practice, and loads of doctors find it really difficult to do proper medication reviews. So there's been loads of positive stuff, working on the QOF [Quality Outcomes Framework]… improving records, liaising with the hospital, liaising with our chemists. So it's hugely beneficial”. (SEL FP VTS, GP supervisor 2)*

Whilst learners’ contributions were valued, sustainability of CoPs was seen as a challenge, with GP supervisors in particular having reservations regarding the amount of time invested in training for a one-off placement.

*“When someone comes in and then they are not replaced that makes it a bit difficult. So, it’s a fine balance in actually giving someone independent practice work, and then, once they’re gone, if there’s not anyone coming afterwards there’s a big gap that you kind of have to fill, […]. Certainly, we have foundation doctors in four months, but they carry on rotating, so, you kind of have them continuously, so you can kind of build your service around them…” (SEL FP VTS, supervisor 1, GP)*

## Discussion

Drawing on qualitative data from two government funded programs this paper explored pharmacy trainees’ and their supervisors’ views of general practice placements and uniquely applied educational theory. Using ’communities of practice’ (CoP) as an interpretive framework enabled a multi-faceted understanding of the mechanisms facilitating learners’ acquisition of skills and knowledge in general practice from a sociocultural perspective. A study limitation was potential self-selection, making findings more positive. Self-reporting, social desirability and recall bias are also other possible limitations. Another limitation was having one researcher code the data. Nonetheless, interpretation of findings was reviewed and agreed between all authors. Any potential bias that could occur due to two of the authors being pharmacists was mitigated due to both co-authors being very experienced health services researchers, and one being a social scientist with particular experience in education theory and research.

Participants considered general practice placements favourably overall and drew out important elements which make an effective CoP. A positive general practice placement experience was confirmed to be intricately tied to effective supervision and a supportive workplace culture. Hallmark features of an effective CoP were provision of authentic patient learning experiences supported by the use of pre- and de-briefing with supervisors [5]. Legitimate peripheral participation [18] allowed for gradual development of competence, with learners initially supervised directly, with supervisors supporting gradual increases in responsibility as confidence and competence increased. Trust was an important mechanism underpinning an effective supervisory relationship, enabling learners to move from peripheral to full CoP participation [30], with learners working more independently, seeking feedback when appropriate.

To aid the development of higher level patient care capabilities, such as complex medication reviews, supervisors employed debriefing, as used with medical students [31]. Vygotsky has described this method as providing ‘scaffolding,’ [32] which helps learners to identify what is needed to conduct a review, through protected time with their supervisors afterwards, to reflect and build on their experience [33].

Kolb’s theory of experiential learning argues that students “learn by doing”, supported by both formal and informal reflection which involves learners stepping back from their experiences and deliberatively interpreting the meaning of their experiences and to plan further experiential learning actions [4]. Learners in our studies benefitted from having access to their supervisor(s) and other general practice staff for support during their day-to-day practice (informal learning), whilst also having protected time with their supervisors to reflect and discuss learning needs(formal learning) [1].

Our findings also highlight the need for guidance on how/when to use workplace-based assessments (WBAs) for pharmacy learners. Evidence from medicine [34] has shown WBAs to be beneficial when used to promote active learning, accompanied by feedback. In our studies, however, WBAs were either used retrospectively without supervisor input, or not at all. Completion of a stipulated number of supervisor supported WBAs could help identify learners’ strengths and weaknesses in performance [35]. Their use would further contribute to consistency across different settings/ communities of practice and provide a mechanism for monitoring progress from peripheral to full participation.

Our findings further indicate that formal educational arrangements such as interprofessional case-based learning in conjunction with collaborative practice, should be considered as a teaching strategy for all learners when designing experiential learning placements [36]. Consistent with the medical education literature [36, 37], case-based learning via MDT meetings was a valuable pedagogical method for interprofessional learning and helped reinforce novice pharmacists’ development of patient consultation capabilities.

The learning approaches reported in this paper are common to both the clerkship and residency phases in medical education; in particular the approach to supervision and the use of pre- and de-briefing [5]. Our findings are consistent with studies investigating general practice placements for medical students and foundation year doctors, where GP supervisors being enthusiastic and committed; learners being actively involved with consultations, gradually taking on more responsibility, and building confidence, are factors contributing to a positive learning environment [32, 38]. This evidence from medical education confirms the validity of our findings, which can inform the design of work-based learning opportunities in different settings and systems/countries. Our findings not only confirm but add to studies on experiential learning for pharmacists in other countries with similar advancements (e.g. Canada, US, Australia) by providing insights into how work-based learning, support and supervision in a CoP facilitates learner development. Hence by using CoP as a theoretical lens, our study formed a supervision model with universal values which could be used for comprehensive planning of experiential learning placements internationally.The findings are particularly relevant to the recently published regulatory standards for the initial education and training of pharmacists in Great Britain and their focus on practice-based learning [39].

The influences of workplace experiences on healthcare students’ career intentions is well recognised in the medical literature [40–42]. Early exposure to general practice, role models, and scope of practice have been shown to contribute to healthcare students selecting careers in general practice [32]. In light of increasing numbers of pharmacists working in general practice under the NHS Long Term Plan, early pharmacy placements in general practice may encourage pharmacists to consider roles in general practice from early in their careers.

## Conclusions

Employing an education theory lens, specifically that of Communities of Practice, this paper identified key factors which contribute to a positive and effective general practice placement learning experience. These insights can usefully inform implementation of work-placed learning to support clinical and patient-centred skills development. Furthermore, pharmacy learners in any setting (including community and hospital pharmacy) need to be provided with protected time and opportunities for meaningful, authentic and supported patient-facing workplace learning experiences, with opportunities for inter professional learning.

## Supplementary Information

**Additional file 1.**

**Additional file 2.**

## Data Availability

The datasets generated and/or analysed during the current study are not publicly available due to protection of participant confidentiality. To request the data, please contact the corresponding author.

## Abbreviations

**CoP**: Communities of practice
**MPharm**: Master of pharmacy
**MDT**: Multi-disciplinary team
**NHS**: National Health Service
**WBAs**: Work-based assessments
