# Supplementary figures and images for: Using communities of practice as a lens for exploring experiential pharmacy learning in general practice: Are communities of practice the way forward in changing the training culture in pharmacy?
Source: BMC Med Educ. 2022 Jan 3;22:12. doi: 10.1186/s12909-021-03079-8 (PMC8722087; doi:10.1186/s12909-021-03079-8)

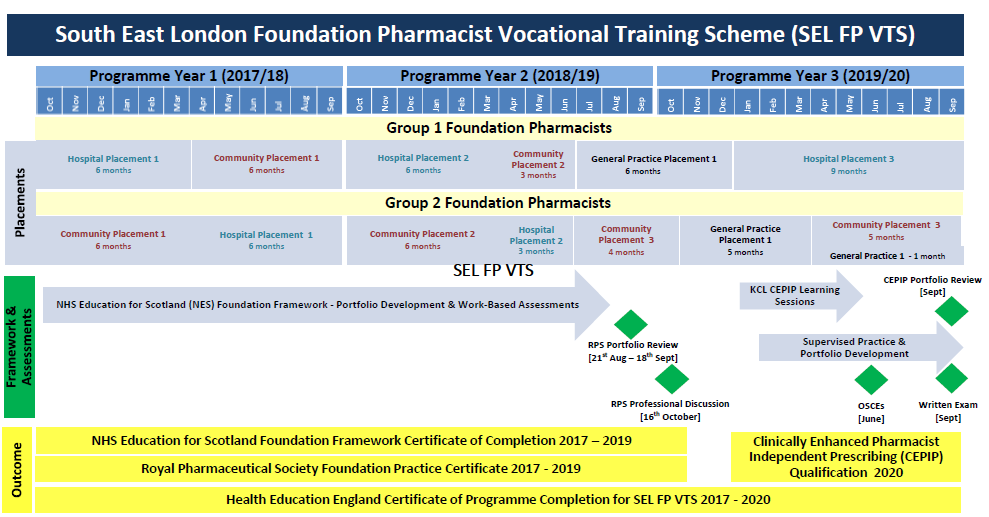


Additional File 1: Summary of the SEL FP VTS programme plan

Supplement: Supplementary file 1 — Additional file 1. [file 12909_2021_3079_MOESM1_ESM.docx]
